# Transforaminal Endoscopic Lumbar Discectomy with versus without Platelet-Rich Plasma Injection for Lumbar Disc Herniation: A Prospective Cohort Study

**DOI:** 10.1155/2022/6181478

**Published:** 2022-03-07

**Authors:** Yi Jiang, Rujun Zuo, Shuai Yuan, Jian Li, Chang Liu, Jiexun Zhang, Ming Ma, Dasheng Li, Yong Hai

**Affiliations:** ^1^Department of Orthopedics, Beijing Chao-Yang Hospital, Capital Medical University, GongTiNanLu 8#, Chao-Yang District, Beijing 100020, China; ^2^Department of Orthopedics (Minimally Invasive Spine Surgery Branch), Beijing Haidian Hospital (Haidian Section of Peking University Third Hospital), ZhongGuanCun Street 29#, Hai-Dian District, Beijing, China; ^3^Department of Radiology Department, Beijing Haidian Hospital (Haidian Section of Peking University Third Hospital), ZhongGuanCun Street 29#, Hai-Dian District, Beijing, China

## Abstract

**Objective:**

Transforaminal endoscopic lumbar discectomy (TELD) is an effective treatment for patients with lumbar disc herniation (LDH) with failure of conservative treatment. However, defects in the annulus fibrosus after TELD usually lead to a recurrence of LDH. Platelet-rich plasma (PRP) injection has shown promising potential for the repair of injured tissues. The combination of TELD and PRP injection has rarely been reported. Hence, this study aimed to evaluate the effectiveness, disc remodeling, and recurrence rate of LDH in TELD with or without PRP in LDH treatment.

**Methods:**

A total of 108 consecutive patients who underwent TELD were prospectively registered between July 2018 and December 2019 (https://clinicaltrials.gov/ct2/show/ChiCTR1800017228). Fifty-one and fifty-seven patients underwent TELD with PRP injections and TELD only, respectively. The visual analog scale (VAS) score for back and leg pain, Oswestry Disability Index (ODI), and MacNab criteria were evaluated, and perioperative complications were documented. The disc protrusion, spinal cross-sectional area (SCSA), and disc height were measured on MRI and evaluated preoperatively, postoperatively, and at regular follow-up.

**Results:**

All patients were followed up. Clinical improvement was noted in both groups. There were statistical differences in the VAS scores of back and leg pain and ODI between the two groups at 3 months, 6 months, and 1 year follow-up (*P* < 0.05); the improvement in the PRP group was significant. The disc protrusion and SCSA on MRI in the PRP group showed better improvement, with lower recurrence rate, than that in the control group at the final follow-up (*P* < 0.05). No adverse events were reported in our study following PRP injection.

**Conclusion:**

Our study showed that TELD with PRP injection was a safe and effective treatment for patients with LDH in the medium and long-term follow-up. PRP injection was beneficial for disc remodeling after endoscopic discectomy and decreased the recurrence of LDH.

## 1. Introduction

Transforaminal endoscopic lumbar discectomy (TELD) is currently an effective and common treatment for patients with lumbar disc herniation (LDH) for whom conservative treatment is ineffective [1, 2]. TELD is characterized by a smaller incision, less blood loss, reduced tissue damage, and shorter hospitalization compared to traditional open surgery [1]. Despite marked improvement in symptoms, intraoperative nerve-related complications are not rare, and numbness and pain after surgery due to irritation of nerve structures may occur with this procedure [2, 3].

Endoscopic discectomy involves removing the prolapsed nucleus pulposus and protruding annulus fibrosus (AF) to free the compressed nerve roots. Liberal removal of intervertebral discs can reduce postoperative radiating pain in the lower limbs, but the defect in the AF after aggressive discectomy may lead to an increase in the recurrence rate and leave patients at risk of potential disability. The defect left after intervertebral disc removal heals by scar tissue formation that can cause fibrosis on the nerve surface; thus, disc remodeling that occurs after the removal of the herniated nucleus pulposus will influence the clinical outcome of the procedure [4]. To improve the clinical efficacy and reduce the recurrence rate, many studies have focused on repairing AF defects with mechanistic devices; however, such devices cannot be used under in a fully endoscopic system because of size limitations [5, 6]. Hence, an increasing number of researchers have focused on biological remediation rather than mechanical repair [7, 8].

Platelet-rich plasma (PRP) application in treating degenerative disc disease and AF repair in in vitro or animal experiments has yielded promising results [9, 10]. As an autologous derivative of whole blood containing a supraphysiological concentration of platelets, PRP can deliver biomolecules to the target injured tissue by modulating inflammatory processes, thereby promoting healing and repair [11]. PRP can also be beneficial in stimulating regeneration by releasing growth factors and proteins that may be involved in repairing the matrices of degenerative discs [12]. Several clinical studies have shown that mild radiculopathy and low back pain due to herniated nucleus pulposus can be improved by percutaneous PRP injection [13, 14]. A recent systematic review and meta-analysis by Xuan et al. [15] assessed PRP injection in the treatment of low back pain and showed an important ability to provide pain relief and patient satisfaction; PRP injection is recommended in the treatment of low back pain with caution due to puncture-related and drug-related complications. To our knowledge, only a few studies have evaluated the safety and effectiveness of TELD combined with PRP for patients with LDH [16] and the current evidence on the treatment of LDH with TELD and PRP is relatively low. The purpose of this study was to prospectively assess the efficacy and safety of PRP injection in patients after TELD and to identify whether PRP injection could provide better clinical efficacy, improve disc remodeling, and decrease the recurrence of LDH.

## 2. Materials and Methods

### 2.1. Study Design

This prospective cohort study included patients who underwent TELD with or without PRP injection from July 2018 to December 2019. The study was conducted in accordance with the principles of the Declaration of Helsinki.

The inclusion criteria were as follows: (1) single-level involvement with symptoms of low back pain and leg pain matched with MRI data, (2) age ranging from 30 to 60 years old, (3) failed conservative treatment after 8 weeks, and (4) platelet count >150 × 10^9^/L. The exclusion criteria were as follows: (1) inability to complete follow-up, (2) LDH with calcification, (3) LDH with lumbar instability, (4) previous lumbar surgery history, (5) cauda equina syndrome, and (6) pregnancy and presence of other comorbidities that could affect coagulation. Participants were allocated into two groups according to the willingness of the patients preoperatively. The PRP group underwent TELD with PRP injection, while the control group underwent TELD only. This study was approved by the Institutional Review Board of Beijing Haidian Hospital (2018002) and registered in the Chinese Clinical Trial Registry (https://clinicaltrials.gov/ct2/show/ChiCTR1800017228). All patients were informed of all possible results of these two surgeries and provided written informed consent preoperatively. The type of operation was selected according to each patient's preference before signing consent.

One hundred and eight consecutive patients were enrolled in this study. Of these patients, 51 underwent TELD combined with PRP injection and 57 underwent TELD only. All patients were followed up for two years to observe clinical efficacy, disc remodeling, and whether there was recurrence of LDH in the surgical segment.

### 2.2. PRP Preparation

**Figure 1:**
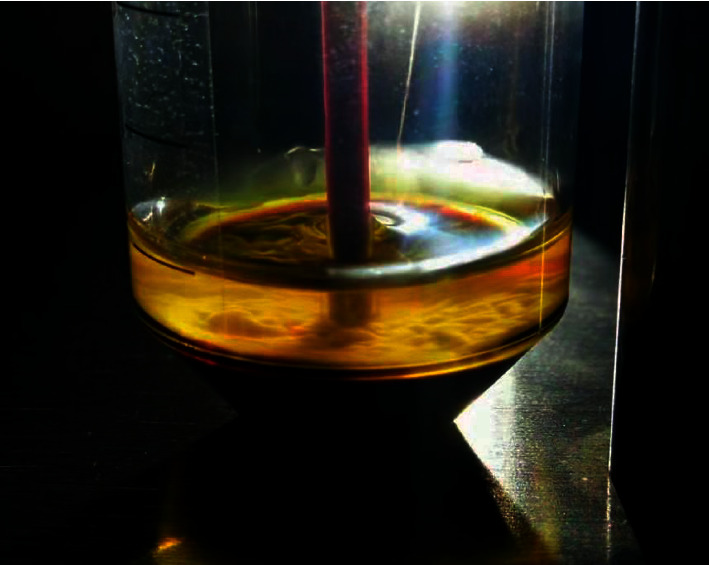
Platelet-rich plasma after the second centrifugation.

PRP injection was performed using a sterile WEGO PRP preparation kit (Wego New Life Medical Devices Co., Ltd., Shandong, China). First, an anticoagulant was extracted using a 50 mL syringe, which sufficiently lubricated the inner wall of the syringe. Whole blood was collected from the median cubical vein the day before surgery. Subsequently, the blood was centrifuged at 2500 rpm for 10 min to separate the whole blood and form a buffy coat (BC) layer containing platelets and white blood cells. Then, a 20 mL syringe was used to remove excess red blood cells 1 mm below the cone. Finally, a second centrifugation was conducted at 2750 rpm for 10 min to further clarify the BC layer. The supernatant was removed using a 20 mL syringe. The pellet contained PRP (Figure 1) and was freshly prepared before use. The PRP volume for the injection was 4 mL.

### 2.3. Surgical Procedure

**Figure 2:**
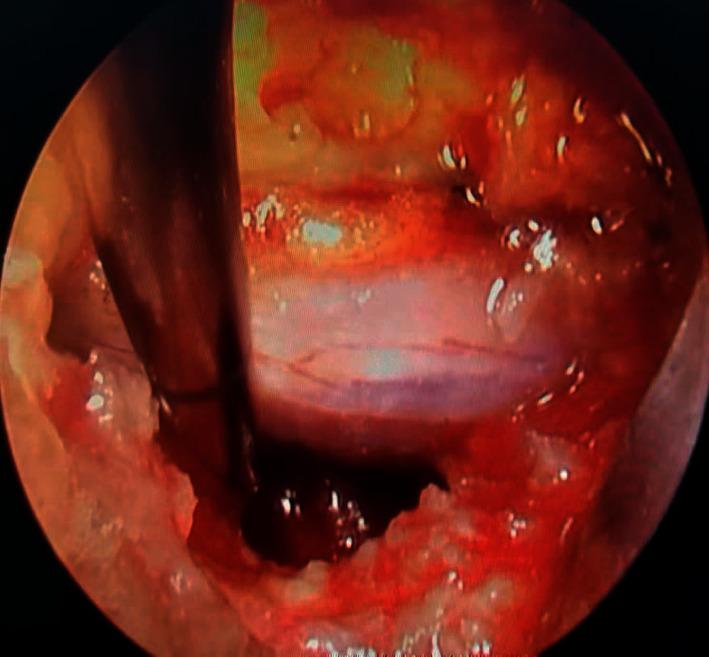
Platelet-rich plasma injection after transforaminal lumbar endoscopic discectomy.

All procedures were performed under local anesthesia administered by the same experienced orthopedic surgeon using the standard technique. Patients were placed in the prone position. The lumbar segment was confirmed using C-arm fluoroscopy. An 18G needle was introduced from the skin entry point to the superior articular process (SAP) of the lower involved vertebral body under fluoroscopic monitoring. After the needle was positioned at the SAP, a guide wire was inserted and a 7 mm incision was made along the guide wire using a #10 scalpel blade. Under the guidance of a guide wire, a series of dilators were introduced sequentially, and a trephine was subsequently inserted through the cannula for foraminoplasty. When the foramen was sufficiently prepared for the working channel, the endoscope was introduced to observe the relationship between the nerve root and herniated nucleus pulposus under continuous irrigation. After removal of the extruded or sequestrated fragment, an annuloplasty was performed to remove the bulging annulus underneath the nerve root and excessive resection was not required, and complete relief of the nerve root was confirmed endoscopically. After draining out the fluids, the fresh PRP was mixed with 0.4 mL thrombin solution (1 : 10) to activate the platelets, which simultaneously yielded a gel. This PRP gel mix was injected into the local site where annuloplasty was performed under endoscopic monitoring in the PRP group (Figure 2). In the control group, discectomy was performed without PRP injection. The incision was closed without drainage in both groups.

### 2.4. MRI Technique

Lumbar spine imaging was performed using a 1.5 Tesla MRI machine (HD-xt, GE Healthcare, USA). The postoperative MRI parameters were turbo echo T2-weighted (T2W) with a 3000 ms repetition time (TR) and 75–85 ms echo time (TE). Axial imaging was set with a slice thickness of 4 mm and an interslice gap of 0.4 mm, 180 × 180 mm field of view (FOV), and a 400 × 288 matrix. Sagittal imaging was performed with a slice thickness of 3 mm without a gap, 280 × 280 mm FOV, and a 400 × 312 matrix.

### 2.5. Measurements

During the perioperative and follow-up periods, demographic characteristics, platelet levels, and perioperative complications, including recurrence, were collected and well documented. For clinical evaluation, each patient completed a questionnaire consisting of standardized outcome assessments. We evaluated clinical outcomes using visual analog scale (VAS) scores for low back pain and leg pain at baseline and at 3 days, 3 months, 6 months, and 1 year after surgery. Oswestry Disability Index (ODI) scores were measured preoperatively and at 3 months, 6 months, and 1 year after surgery. Patient satisfaction was graded as excellent, good, fair, or poor using the MacNab criteria.

**Figure 3:**
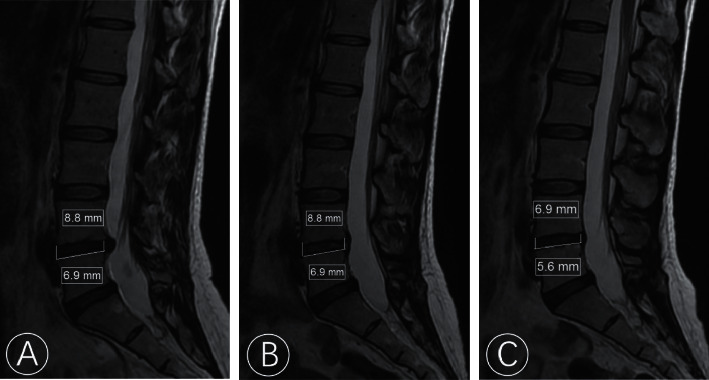
Comparison of disc height (DH) on MRI preoperatively (a), at 3 days (b), and at 1 year after operation (c) with the upper number which represents the anterior DH and the lower number represents the posterior DH.

**Figure 4:**
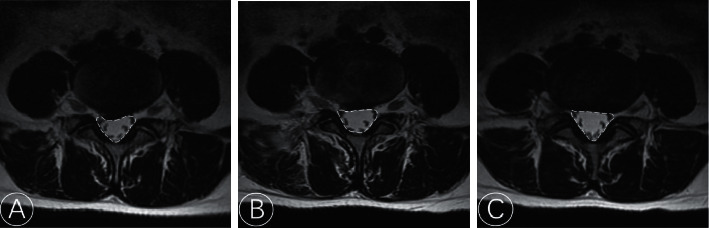
Comparison of spinal cross-sectional area (SCSA) on MRI preoperatively (a), at 3 days (b), and at 1 year after operation (c).

**Figure 5:**
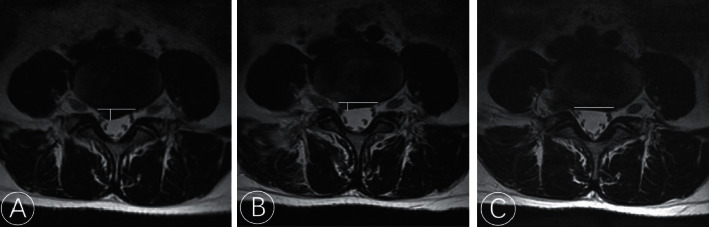
Comparison of disc protrusion size on PRP group preoperatively (a), at 3 days (b), and at 1 year after operation (c), which was measured by drawing a line at base of the disc and then a perpendicular line was drawn from the first line to the most protruded point on MRI.

Radiological parameters were obtained on MRI images before surgery, 3 days after surgery, and at the last follow-up. The disc height (DH) was computed as the mean of the anterior and posterior DH (Figure 3). Spinal cross-sectional area (SCSA) was calculated on the axial cut of the T2W image using miPlatform 3.0 software (Hinacom Software and Technology, Beijing, China) (Figure 4). Disc herniation grade was classified according to the Michigan State University (MSU) classification. Disc protrusion size was measured in the axial cut of the MRI where the disc was most prominent (Figure 5).

Degeneration of the intervertebral disc was graded using the Pffirmann grade classification. All parameters were calculated separately by one spine surgeon and one radiologist.

### 2.6. Statistical Analysis

Measurement data are expressed as the mean ± standard deviation. Categorical variables were presented as frequencies and percentages. A *t*-test was used for parametric data, and the chi-squared test was used for categorical variables. Interobserver reliability was calculated using the intraclass correlation coefficient (ICC). The analysis was performed using SPSS (version 22.0; IBM Corp., Armonk, NY, USA). Statistical significance was set at *P* < 0.05.

## 3. Results

All patients completed 1 year of follow-up. No significant differences in baseline characteristics were noted between the two groups (Table 1).

Comparison of VAS scores for back and leg pain before and after surgery in each group revealed no statistical difference (*P* > 0.05). On the contrary, VAS scores for back and leg pain were significantly lower in the PRP group at the 3 months, 6 months, and 1 year follow-up (*P* < 0.05) than in the control group, suggesting better postoperative recovery of the patients. The corresponding ODI in the PRP group at the 3 months, 6 months, and 1 year follow-up was also significantly lower than in the control group (*P* < 0.05), indicating that the PRP group achieved better life function improvement. The good and fair rate of the MacNab criteria was 90.2% (46/51) in the PRP group and 89.5% (51/57) in the control group, which was not significantly different (Table 2).

Residual annulus, observed via MRI, is common in the early postoperative period after TELD and gradually decreases over time. The disc protrusion could still be observed postoperatively, although the pain may have improved dramatically because of the decompression around the nerve root. The disc protrusion size was reduced in the last follow-up MRI compared to the postoperation MRI, and this was more significant in the PRP group than in the control group. The increased postoperative SCSA indicates that the endogenous repair is connected to the residual annulus. In our study, the SCSA increased from 174.01 ± 43.32 mm^2^ to 218.95 ± 32.80 mm^2^ in the PRP group and from 165.11 ± 31.51 mm^2^ to 201.15 ± 49.16 mm^2^ in the control group. The degree of change was significantly higher in the PRP group than in the control group (*P* < 0.05). Mean DH was 10.15 ± 3.77 mm and 8.85 ± 3.48 mm at 3 days and 1 year after surgery in the PRP group, respectively, and 9.97 ± 3.34 mm and 8.64 ± 3.20 mm in the control group with no significant difference (*P* > 0.05) (Table 3). The comparation of Pffirmann grading and MSU classification preoperatively and at 3 days and 1-year follow-up showed no significant difference, respectively (*P* > 0.05). Inter-rater reliability between the two observers was calculated using ICC. The ICC of DH and SCSA was 0.826–0.975, indicating excellent reliability.

### 3.1. Complications

Five patients underwent revision surgery due to recurrence. Of these patients, 1 patient was in the PRP group, and 4 patients were in the control group. The recurrence rate was 1.96% (1/51) in the PRP group, which was significantly lower than the 7.02% (4/57) in the control group (*P* < 0.05). No puncture-related or drug-related complications or damage to the exiting nerve root, traversing nerve root, or dura mater occurred.

## 4. Discussion

This is a prospective cohort study comparing the clinical efficacy and safety, disc remodeling, and recurrence rate of TELD combined with PRP injection and TELD alone for the treatment of LDH [2]. The present results demonstrated that the treatment of LDH by TELD combined with PRP injection and TELD alone could achieve satisfactory clinical and radiological results. However, VAS and ODI scores at follow-up were significantly better in patients treated with PRP injections. PRP injection could promote the disc remodeling process after endoscopic discectomy and prevent recurrence in the residual disc. There were no signs of segmental instability, muscle weakness, paresthesia, or cauda equina syndrome on radiographic and clinical examinations in all patients.

With an increase in desk work hours in modern life, the incidence rate of LDH is increasing year by year, accompanied by a trend of occurrence in younger age [17]. LDH leads to radiating pain caused by compression of nerve structures, and decompression is critical for treatment. Although the operation can contribute to good decompression, pain, and paresthesia reduction after surgery, it still affects the quality of life of patients because of inflammation from various causes [18]. Local inflammation factors, as one of the primary causes of pain, should not be ignored. To further improve the efficacy, PRP injection after discectomy can not only improve the clinical effectiveness but also repair the annular fibrosis [16]. The diverse factors released by PRP have positive effects on cell survival and proliferation, downregulation of proinflammatory cytokines, suppression of *κ*B pathways induced by tumor necrosis factor and interleukin-1*β*, endogenous cannabinoid systems, subchondral bone homeostasis, and bone mineralization [19, 20]. Kamoda et al. [21] reported that PRP could reduce the inflammatory calcitonin gene-related peptide in sensory neurons innervating the discs. Perineural PRP injection could promote improvement in peripheral neural function by decreasing the proportion of inflammatory neuropeptides [22]. Many researchers have revealed that PRP injection is effective in treating LDH and can maintain long-term clinical results [23–25]. The aforementioned studies suggested that the effectiveness of PRP injection was not only related to the interaction of local inflammatory factors but also to the repair of local damage. In our study, the patients who underwent PRP injection showed greater improvement in pain relief, and postoperative pain or numbness could be reduced with PRP; this finding was equivalent to the findings of previous trials. We suggest that PRP injection around the lesion may release many factors that could reduce inflammation and improve symptoms. Furthermore, there was a significant difference in the ODI score between the two groups in our study. This might be caused by the various factors released from PRP, which may play a role in interfering with scar formation around the nerve structure. The effects of PRP infiltration on extracellular matrix synthesis, anti-inflammatory mechanisms, analgesia, and subchondral bone homeostasis may contribute to disc restoration. All of these could stimulate the endogenous repair machinery and improve function.

Discectomy inevitably leads to further degeneration of the intervertebral disc. We observed that both the Pffirmann classification and the MSU classification revealed that the intervertebral disc underwent degenerative changes after surgery in both groups, but there was no significant difference between the two groups. DH loss is a natural process of degeneration after discectomy. A previous study reported that PRP injection could prevent a decrease in DH in a rabbit model [26]. However, in our series, DH decreased in both groups at one year, with no significant difference. We suggest that there is a difference in movement characteristics between animal models and human beings. These different characteristics can lead to different results.

Residual annulus observed via MRI is common in the early postoperative period after TELD. The changes in SCSA and the degree of disc herniation over time suggest that the disc has the potential for remodeling [27]. In our study, the SCSA and disc protrusion were smaller in the PRP group during follow-up, which indicates that PRP plays a positive role in the remodeling of the annulus fibrosus. These changes result in an expansion of the spinal canal space and reduced compression and irritation of the nerve roots, which accounts for the improvement of clinical symptoms from another aspect. PRP has proliferative effects on chondrocyte-like cells in the anterior inner AF which could turn fibrotic tissue into cartilaginous tissue which will decrease scar formation and can increase extracellular matrix production in vitro [28, 29]. PRP supplementation created a gel-like structure, which affected the morphology of the disc cells; therefore, it may be considered to promote disc repair [30]. The doctor can evaluate the patient's current condition and provide correct guidance by observing disc remodeling and combining it with clinical improvement. The recurrence rate was significantly lower in the PRP group in our study, indicating that PRP can help prevent recurrence. Ideal remodeling of the AF can effectively set up a mechanical barrier to prevent further recurrence of disc herniation, reduce the occurrence of recompression and expand the area of the spinal canal [16], avoid postoperative intraspinal fibrotic scar formation, and minimize the release of inflammatory factors, thereby improving clinical efficacy and avoiding the occurrence of postoperative pain and numbness. After appropriate AF repair, the intervertebral disc may be seen as a closed space that prevents the leakage of various biological agents; this provides the basis for the biological treatment of intervertebral discs, and homeostasis of the intervertebral disc is the basis for avoiding recurrence [16].

In our study, we compared the clinical efficacy and safety, disc remodeling, and recurrence rate of TELD combined with PRP injection and TELD alone for the treatment of LDH. As we expected, PRP injection produced better clinical effects, affected disc remodeling positively, and prevented disc recurrence. The complication rate was low; there were five cases of recurrence, one in the PRP group, and four in the control group. No cases of nerve-related injuries occurred in the present study.

This study had some limitations. Nonrandomization may have caused interference in the analysis of the results. The orientation of the axial cut of the T2W image at one year was not the same with that in the previous MRI. Measurement bias was inevitable, although two independent researchers were involved in this study to improve the accuracy of the measurements. Finally, the sample size in this study was small. Hence, further investigation with a larger sample size and long-term observation is needed.

## 5. Conclusions

Our study showed that TELD with PRP injection was a safe and effective treatment for patients with LDH in the medium and long-term follow-up. PRP injection was beneficial for disc remodeling after endoscopic discectomy and decreased the recurrence of LDH.

## Figures and Tables

**Table 1:** Patient demographics, presentation, and procedural information (mean ± SD).

Characteristics	PRP group, *n* = 51	Control group, *n* = 57	*P* value
Age	48.1 ± 10.25	45.9 ± 9.83	0.257
*Sex*			
Male	33 (57.9%)	32 (62.7%)	0.607
Female	24 (42.1%)	19 (37.3%)	
*Levels*			0.388
L3/L4	6 (11.8%)	7 (12.3%)	
L4/L5	33 (64.7%)	30 (52.6%)	
L5/S1	12 (23.5%)	20 (35.1%)	
Platelet levels (×10^9^/L)	217.0 ± 52.1	235.9 ± 65.9	0.225
*Pfirrmann grading*			0.391
Grade 2	9 (17.6%)	5 (8.8%)	
Grade 3	34 (66.7%)	42 (73.7%)	
Grade 4	8 (15.7%)	10 (17.5%)	
*MSU-herniated disc grade*			0.939
Grade 1	13 (25.5%)	16 (28.1%)	
Grade 2	29 (56.9%)	32 (56.1%)	
Grade 3	9 (17.6%)	9 (15.8%)	
*Preoperative VAS score*			
Leg pain	7.19 ± 2.34	6.80 ± 2.15	0.373
Back pain	5.02 ± 2.55	4.74 ± 3.19	0.612
Preoperative ODI (%)	50.85 ± 19.14	49.73 ± 25.89	0.802

SD, standard deviation; VAS, visual analog scale; ODI, Oswestry Disability Index; MSU, Michigan State University classification system; PRP, platelet-rich plasma.

**Table 2:** Comparison of clinical outcomes at 3 days, 6 months, and 1 year after operation (mean ± SD).

Characteristics	Control group, *n* = 57	PRP group, *n* = 51	*P* value
*VAS score*			
Leg pain at 3 days	1.54 ± 1.60	1.69 ± 1.55	0.641
Back pain at 3 days	1.45 ± 1.10	1.68 ± 1.91	0.446
Leg pain at 3 months	1.70 ± 0.91	1.33 ± 0.80	0.027^*∗*^
Back pain at 3 months	1.35 ± 0.97	0.96 ± 0.63	0.016^*∗*^
Leg pain at 6 months	1.79 ± 0.90	1.39 ± 0.83	0.019^*∗*^
Back pain at 6 months	1.23 ± 0.84	0.86 ± 0.63	0.012^*∗*^
Leg pain at 1 year	1.09 ± 0.93	0.75 ± 0.74	0.038^*∗*^
Back pain at 1 year	1.11 ± 0.82	0.82 ± 0.56	0.041^*∗*^
*ODI (%)*			
3 months	14.10 ± 9.99	10.62 ± 6.53	0.037^*∗*^
6 months	10.80 ± 10.99	6.65 ± 6.51	0.021^*∗*^
1 year	6.17 ± 4.47	4.29 ± 4.51	0.031^*∗*^

SD, standard deviation; VAS, visual analog scale; ODI, Oswestry Disability Index; PRP, platelet-rich plasma, ^*∗*^*P* < 0.05.

**Table 3:** Comparison of MRI features at 3 days and 1 year after operation.

Characteristics	Control group, *n* = 57	PRP group, *n* = 51	*P* value
*DH (mm)*			
3 days	9.97 ± 3.34	10.15 ± 3.77	0.222
1 year	8.64 ± 3.20	8.85 ± 3.48	0.350
*SCSA (mm* ^2^)			
3 days	165.11 ± 31.51	174.01 ± 43.32	0.230
1 year	201.15 ± 49.16	218.95 ± 32.80	0.031^*∗*^
*Pfirrmann grading*			0.751
Grade II	1 (1.8%)	2 (3.9%)	
Grade III	36 (63.2%)	30 (58.8%)	
Grade IV	20(35.1%)	169 (37.3%)	
*MSU-herniated disc grade*			0.461
Grade 1	40 (70.2%)	39 (76.5%)	
Grade 2	17 (29.8%)	10 (23.5%)	
*Disc protrusion (mm)*			
3 days	3.23 ± 1.28	3.34 ± 1.36	0.680
1 year	2.19 ± 0.96	1.77 ± 1.10	0.043^*∗*^

DH, disc height; SCSA, spinal cross-sectional area; PRP, platelet-rich plasma; MRI, magnetic resonance imaging, ^*∗*^*P* < 0.05.

## Data Availability

The experiment data used to support the findings of this study are available from the corresponding author upon request.
